# Reliability and validity of the Sheehan disability scale modified for pathological gambling

**DOI:** 10.1186/1471-244X-13-177

**Published:** 2013-07-01

**Authors:** David C Hodgins

**Affiliations:** 1 Department of Psychology, University of Calgary, 2500 University Drive NW, Calgary, AB T2N 1N4, Canada

**Keywords:** Functional Impairment, Pathological Gambling, Sheehan Disability Scale, Clinical Trials

## Abstract

**Background:**

An interview format version of the Sheehan Disability Scale (SDS) modified to assess gambling treatment outcomes was assessed for reliability and validity. The SDS assesses impairment in work, family and social functioning related to mental disorders.

**Methods:**

A pilot study (N = 21) determined the preferred wording for oral administration. Participants with pathological gambling in a relapse prevention clinical trial completed the scale and other validation measures at baseline, six and twelve month follow-ups (N = 169).

**Results:**

Confirmatory factor analysis supported a one factor solution and the scale had good internal reliability for a three item scale. The SDS was sensitive to change and correlations with recent gambling behaviour and severity were moderate as expected. Similarly, correlations with self-efficacy, perceived control over gambling, and craving were moderate, but they were lower for less directly related constructs such as depression and perceived family and friend support.

**Conclusions:**

The SDS is a brief, psychometrically sound, outcome measure of impairment associated with gambling disorders that can be administered by telephone.

## Background

The Sheehan Disability Scale (SDS) was originally designed to assess functional impairment associated with anxiety disorder diagnoses [1]. Functional impairment or disability is related to, but conceptually distinct from, symptom severity and distress. The SDS is a three-item self-completion scale measuring the impact of symptomatology on work, social and family functioning. Because of its simplicity, brevity, and high face validity, and because of the lack of competing brief measures of functional impairment, the SDS has become a widely used outcome measure in clinical trials of a variety of mental health disorders. The SDS has been used for obsessive compulsive disorder, post-traumatic stress disorder, major depressive disorder, panic disorder, social anxiety disorder, schizophrenia [2] and, most recently, pathological gambling [3-6]. As of 2007, the SDS had been translated from English into 48 languages [2].

Despite its popularity, limited data on the psychometric properties of the SDS have been published. Leon, Shear, Portera and Klerman [7] analyzed clinical trial data obtained from patients with panic disorder. The authors reported that the scale had adequate internal reliability (α = .56 to .86) and a stable one factor structure at baseline and post-treatment follow-up. They also demonstrated that the measure is sensitive to treatment-related change and that the scores, as expected, were moderately associated with panic symptoms. Leon and colleagues [8] also examined the SDS in a sample of primary care outpatients concurrently assessed for mental health disorders. Internal reliability was excellent (α = .89) and a one factor structure was also supported. In terms of validity, patients who met the criteria for a mental disorder had more impairment than those who did not. The authors used a cut-point of five or greater on the 30-point scale, and they found that the SDS had good sensitivity and specificity in identifying the mental disorder patients.

Sheehan and Sheehan [2] reviewed the use of the scale in published clinical trials in order to establish interpretation guidelines for patient improvement. They provide preliminary recommendations that a total score of 6 or less indicates remission and a score of 12 or less indicates treatment response. A reduction of approximately 4 points indicates improvement [2].

In the gambling arena, a number of well validated interview and self-rated measures of symptom severity are available [9] including: the South Oak Gambling Screen (SOGS) [10], the Problem Gambling Severity Index (PGSI) of the Canadian Problem Gambling Index [11,12], the National Opinion Research Centre DSM Screen for Gambling Problems (NODS), [13], the Gambling Symptom Assessment Scale [14], and the Gambling Treatment Outcome Monitoring System [15]. Additionally, interview measures of self-reported gambling behaviours (frequency, money and time spent) show good psychometric properties [16,17]. Measures of impairment include the SDS as well as a gambling adaptation of the Addiction Severity Index (ASI-G), which is an interview measure that assesses functioning and need for treatment in a variety of domains including gambling problems, physical health, employment, social relationships, psychiatric functioning, alcohol and other drug use and legal problems. The gambling subscale has been well validated in treatment populations [18-20] whereas the other subscales have been mostly validated with substance abuse populations. The SDS has not been assessed psychometrically despite its use.

The purpose of this report is to provide data on the reliability and validity of an interview version of the Sheehan Disability Scale modified for gambling (SDS-G). Data from a trial of a distance relapse prevention program were used to assessment the psychometric properties. One of the unique features of the SDS is that the scale items are presented visually as a horizontal line marked with both numbers (0 to 10) and verbal anchors (0 = Not at all, 1–3 = mildly, 4–6 = moderately, 7–9 = markedly, and 10 = extremely). The numbers are presented in a larger font than the verbal anchors and respondents are required to choose a specific number to indicate their response. This design is easy for patients to complete, but, unfortunately, limits the scale from being used in a face-to-face or telephone interview format. Because telephone follow-up is often used in gambling treatment studies, the first step in this investigation was to solicit feedback from interviewers and respondents with pathological gambling about the clarity of different response options in an interview format. The response options were modified from the instructions provided for the self-completion version.

## Methods

Ethical approval for this study was provided by the Conjoint Faculties Ethic Review Board at the University of Calgary and all participants provided written informed consent.

### Examination of response options

Two experienced interviewers participated in this pilot examination of response options. The pilot study was conducted to determine the preferred wording for telephone interviews. Participants (N=21) meeting the criteria for pathological gambling, as part of a telephone assessment, were informed by the interviewer that: “I am going to ask you some questions regarding how your gambling problem has affected your life in the past month”. Participants where then asked two questions about each of the three domains. The domains were asked in the standard order used previously (work, social and family), but question response order was randomized. For example: “To what extent has your gambling disrupted your work or studies in the past month? Would you say not at all, mildly, moderately, markedly, or very severely? To what extent has your gambling problem disrupted your work or studies in the past month on a scale of 0 to 10, with zero indicating not at all and 10 indicating severely?” Participant agreement across the two response options was computed and the interviewers provided qualitative feedback on their preferences.

### Psychometric examination

#### *Participants*

For the trial of the distance relapse prevention program, a sample of individuals meeting criteria for pathological gambling (N = 169) was recruited through media announcements and was assessed via telephone [21]. Gambling outcomes included self-reported gambling behaviours, confirmed by collateral reports, and gambling problem severity. Assessments were conducted at baseline, six weeks, six months and twelve months by trained research assistants. Results indicated that extended versus brief support did not improve gambling outcomes over 12 months.

#### *Interview measures*

The initial interview included: a gambling history measure including a timeline follow-back interview assessing gambling behaviour [16]; two measures of gambling problem severity (the NODS, which measures DSM-IV criteria for the past year and lifetime [13] and the SOGS [10]), which measures past year problem severity; a measure of self-efficacy for quitting gambling (Gambling Abstinence Self-efficacy Scale or GASS) [22]; a measure of gambling craving over the past 24 hours [23]; a measure of current depression (Centre of Epidemiologic Studies – Depressed Mood Scale or CES) [24]; and the SDS-G. Based upon the pilot study, the specific questions asked were:

•To what extent has your gambling problem disrupted your work or studies in the past month on a scale of 0 to 10, with zero indicating not at all and 10 indicating extremely?

•To what extent has your gambling problem disrupted your social life in the past month on a scale of 0 to 10, with zero indicating not at all and 10 indicating extremely?

•To what extent has your gambling problem disrupted your Family life/household responsibilities in the past month on a scale of 0 to 10, with zero indicating not at all and 10 indicating extremely?

The six month follow-up interviews included: a timeline follow-back to capture gambling behaviour since the last assessment; the GASS; the SDS-G; and a measure of perceived control over gambling (0–10 point scale). The twelve month assessments included: the instruments used at six months plus the past-year versions of the NODS and SOGS and a third measure of problem severity, the PGSI; the CES; an unpublished six item past week gambling craving measure; and a measure of perceived social support provided by family and friends [25].

#### *Analyses*

Internal reliability was computed for baseline, six and twelve month data. Confirmatory factor analysis, using maximum likelihood structural equation modelling, was computed using six and twelve month data. To maximize sample size, baseline data were excluded and missing data (6 cases at 12 months) were estimated using full information maximum likelihood [26]. Model fit was assessed using the χ^2^/*df* ratio, comparative fit index (CFI) and the root mean square error of approximation (RMSEA). Criteria for good fit was considered to be χ^2^/*df* ratio < 3.00 [27], CFI > 0.95 [28] and RMSEA < 0.05 [29]. Concurrent validity was assessed with Pearson correlations between SDS-G scores and measures of gambling behaviour, problem gambling severity and self-efficacy at each assessment. Sensitivity to change was assessed in two ways. Changes in SDS-G scores over time were examined using one-way repeated measures ANOVA. The association between change in SDS-G scores and change in SOGS, days of gambling and depression from baseline to the 12 month follow-up was examined using Pearson correlations of difference scores. Analyses were conducted using SPSS and AMOS V.19.

## Results

### Examination of response options

Intraclass correlation coefficients showed that the two response options correlated strongly for each of the three domains, work ICC = 0.95, social life ICC = 1.00, family/home responsibilities ICC = 0.94. The two interviewers preferred the 0 to 10 point scale option, indicating that it was easier to administer orally.

### Psychometric examination

A total of 169 participants met the inclusion criteria for this study. The SDS-G was administered after the trial had already commenced; therefore, we did not have baseline data for 113 participants. However, the final 56 participants recruited did complete the SDS-G at baseline. All participants successfully followed at six (n = 146) and twelve months (n = 142) completed the SDS-G. There were no significant differences in baseline demographics or gambling variables between participants who did and did not complete the SDS-G at baseline or participants who were or were not followed at six and twelve months [21].

Participant mean age was 42 years (SD = 11.2, range 21–65) and 42% were female. Most participants reported problems with video lottery terminals (VLTs, 80%), slot machines (38%) and casino games (20%). Participants reported having had a gambling problem since the mean of age 34 (SD = 11.2) and 60% had had previous treatment or had attended Gamblers Anonymous. The mean SOGS total was 11.3 (SD = 3.3) and the mean lifetime NODS score was 8.6 (SD = 1.2).

Table 1 displays means (SD) and the Pearson correlations between the individual items and total scores at each assessment. Internal reliability was estimated at α = 0.56 at baseline, α = 0.78 at six months and α = 0.81 at 12 months. All three values are adequate for a three item scale. The relatively low value for the baseline may be partly due to the smaller sample size.

**Table 1:** Means (SD) and Pearson correlations between individual items and total scores at each assessment

	**Mean**	**SD**	**Range**	**Work**	**Social**	**Family**	**Total**
Initial (N=46)							
Work	1.7	3.0	0-10	-	.17	.33	.62
Social	4.2	3.9	0-10			.40	.75
Family	5.4	3.8	0-10				.81
Total	11.4	7.9	0-30				-
Six months (N = 146)							
Work	1.0	2.5	0-10	-	.54	.48	.78
Social	1.5	2.9	0-10			.63	.86
Family	2.1	3.2	0-10				.86
Total	4.5	7.8	0-30				-
Twelve months (N=142)							
Work	1.00	2.4	0-10	-	.53	.52	.77
Social	1.6	2.8	0-10			.71	.88
Family	2.1	3.3	0-10				.90
Total	4.7	7.2	0-30				-

**Figure 1:**
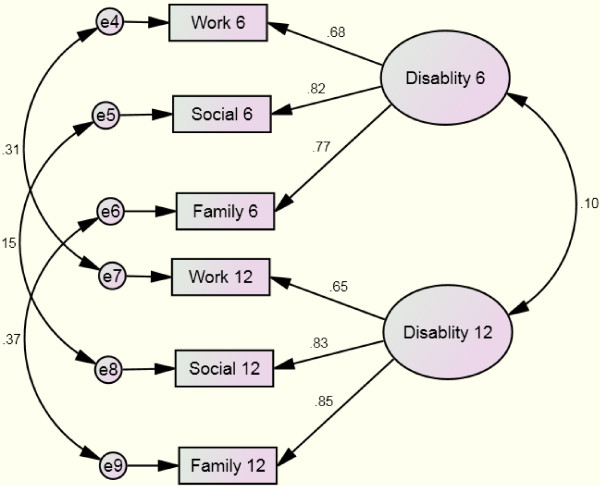
Confirmatory factor structure of the Sheehan Disability Scale for gambling over time.

Figure 1 displays the one factor structure at six and twelve months along with standardized coefficients. The fit indices suggested excellent fit: χ^2^/*df* ratio = 1.30, CFI = 0.99 and RMSEA = 0.044. The work variables loaded slightly less strongly on the latent disability constructs than family and social impairment items.

Table 2 displays Pearson correlations between gambling behaviour and severity measures and measures of a number of other constructs such as depression and social support. Correlations with each of the three SDS-G items as well as the total score are provided. Correlations with recent gambling behaviour and severity were moderate as expected. Similarly, correlations with self-efficacy and perceived control over gambling and craving were moderate. However, correlations with lifetime problem gambling severity (NODS) were non-significant. This measure was obtained at baseline and with a smaller sample size. Finally, SDS items and total correlated with depression and perceived family and friend support.

**Table 2:** Pearson Correlations between SDS-G total and items and external constructs

	**Work**	**Social**	**Family**	**SDS-G**
				**Total**
Initial (N = 56)				
NODS- lifetime	.20_n.s._	.01 _n.s._	.18 _n.s._	.17 _n.s._
NODS – past year	.24 _n.s._	.16 _n.s._	.35	.34
SOGS – past year	.36	.16 _n.s._	.51	.46
Days abstinent – prior to initial	-.13 _n.s._	-.26	-.29	-.31
CES	.15 _n.s._	.30	.20 _n.s._	.30
Six months (N = 146)				
Perceived control	-.47	-.47	-.48	-.58
GASS	-.37	-.50	-.40	-.52
Days gambled – past month^1^	.26	.39	.32	.41
Twelve months (N = 142)				
Perceived control	-.21	-.41	-.44	-.43
GASS	-.21	-.39	-.52	-.46
Days gambled – past month	.26	.57	.49	.52
Craving	.32	.52	.58	.59
NODS – past year	.39	.51	.53	.56
SOGS – past year	.39	.53	.56	.58
PGSI	.35	.48	.50	.52
CES	.34	.40	.50	.51
Family support	-.13 _n.s._	-.22	-.27	-.27
Friend support	-.22	-.27	-.35	-.33

Note. *NODS* NORC DSM Screen for Gambling, *SOGS* South Oaks Gambling Screen, *CES* Centre of Epidemiologic Studies – Depressed Mood Scale, *GASS* Gambling Abstinence Self-Efficacy Scale, *PGSI* Problem Gambling Severity Index. All correlations significant except where indicated (ns=non-significant). ^1^n = 142.

In terms of sensitivity to change, SDS-G total scores for individuals followed at all three assessments showed a significant reduction from baseline (M = 10.5, SD = 8.0) compared to the six (M = 3.1, SD = 5.2) and twelve month follow-up periods (M = 4.4, SD = 7.0; F (2, 41) = 15.3; p<0.0001; partial eta squared = 0.43). Change in SOGS scores, days of gambling, and CES depression scores from baseline to twelve month follow-up correlated with changes in SDS-G scores at r = 0.54, 0.33, and 0.53 respectively (p<0.0001).

## Discussion

These analyses provide evidence in support of a gambling modified SDS to assess gambling treatment outcomes via interview. Because of the nature of the relapse prevention study, this sample of pathological gamblers includes a range of gambling involvements from abstinent to heavy gambling, which is optimal in terms of validating a scale. The results supported a one factor solution and the scale had good internal reliability for a three item scale. The SDS-G appears sensitive to change and it appears to correlate more strongly with other measures of current functioning than lifetime measures.

Future research should assess of retest reliability of the scale and it would also be useful to assess concurrent validity of the very brief scale against more lengthy measures of functional impairment such as the Social Adjustment Scale [30] and the Medical Outcome Study SF-36 [31]; however, these scales have not been validated for pathological gambling samples. Validation of a self-completion version would also be valuable. Moreover, some clinical trial studies report the individual item scores in addition to the total scores. The reliability and validity of individual items needs further examination. In the structural equation model, the work variables loaded less strongly on the latent disability construct than the family and social variables and, similarly, the work variable correlated less strongly with the SDS-G total (see Table 1). The work variable also correlated less strongly with the concurrent validity variables than did the social, family and total scores (see Table 2). This difference may be related to the fact that gambling problems have less impact on work functioning or that preservation of work functioning is important to finance continued gambling (see mean scores on Table 1), or it may indicate a weaker measure of the work disability construct.

Although these analyses support the psychometric strength of this brief scale, they do not provide validated interpretation guidelines for improvement or adequate social functioning. The existing guidelines are not disorder specific and are meant to be used for a variety of disorders. Nonetheless, validation for each disorder individually is crucial. Interpretation guidelines would increase the practical utility of using this scale in outcome studies as well as use by clinicians monitoring patent outcome.

## Conclusions

In conclusion, the SDS-G total score should work well as an outcome measure that is correlated with both gambling behaviour and gambling problem severity, but also conceptually and statistically distinct. Despite its brevity, it shows good internal reliability, good evidence of factorial validity and it is sensitive to patient changes.

## Competing interests

David Hodgins has no competing interests.

## Pre-publication history

The pre-publication history for this paper can be accessed here:

http://www.biomedcentral.com/1471-244X/13/177/prepub
